# Pattern of Palliative Care, Pain Management and Referral Trends in Patients Receiving Radiotherapy at a Tertiary Cancer Center

**DOI:** 10.4103/0973-1075.58462

**Published:** 2009

**Authors:** Kuldeep Sharma, Bidhu K Mohanti, Gaura K Rath, Sushma Bhatnagar

**Affiliations:** Department of Radiation Oncology, Institute Rotary Cancer Hospital, All India Institute of Medical Sciences, New Delhi, India; 1 Department of Anesthesia, Institute Rotary Cancer Hospital, All India Institute of Medical Sciences, New Delhi, India

**Keywords:** Cancer pain, Palliative care, Palliative radiotherapy, Referral pattern

## Abstract

**Background:**

Pain is a common primary symptom of advanced cancer and metastatic disease, occurring in 50-75% of all patients. Although palliative care and pain management are essential components in oncology practice, studies show that these areas are often inadequately addressed.

**Materials and Methods:**

We randomly selected 152 patients receiving palliative radiotherapy (PRT) from October 2006 to August 2008, excluding metastatic bone lesions. Patients' records were studied retrospectively.

**Results:**

A median follow-up of 21 weeks was available for 119 males and 33 females with a median age of 55 years. Maximum (60%) patients were of head and neck cancers followed by esophagus (14%), lung (10%) and others. Dysphagia, growth/ulcer and pain were the chief indications for PRT. Pain was present in 93 (61%) cases out of which, 56 (60%) were referred to pain clinic. All except one consulted pain clinic with a median pain score of 8 (0-10 point scale). Fifty-three of these 56 patients (96%) received opioid-based treatment with adequate pain relief in 33% cases and loss of follow-up in 40% cases. Only five (3%) cases were referred to a hospice. Twenty-two (14%) cases were considered for radical treatment following excellent response to PRT.

**Conclusion:**

In this selective sample, the standard of analgesic treatment was found to be satisfactory. However, there is a lot of scope for improvement regarding referral to pain clinic and later to the hospice. Patients' follow-up needs to be improved along with future studies evaluating those patients who were considered for further RT till radical dose. Programs to change the patients' attitude towards palliative care, physicians' (residents') training to improve communication skills, and institutional policies may be promising strategies.

## INTRODUCTION

Up to 70% of patients with cancer are expected to experience pain during the cancer illness and it is more likely as the disease progresses.[1] Severe pain does not only cause enormous suffering, it also leads to impairment in the activities of daily living, psychological, familial and professional dysfunction and disturbance of sleep, appetite, and vitality. Thus, adequate treatment of pain is a major aim in these patients.[2]

Despite the introduction of the World Health Organisation WHO ladder for pain management more than two decades back, cancer pain is still a major problem in clinical practice.[3] Investigations have shown that 40-50% of patients with cancer pain receive insufficient analgesic therapy despite these guidelines.[2] There are various factors that contribute to these shortcomings.

Several studies have documented under-reporting of symptoms and under-treatment of pain in cancer patients and the most frequently identified barriers in adequate pain management are found to be physician's underestimation and patient's reluctance to report pain.[3] Patients with the same age, gender, and diagnosis can present with different factors having implications for their pain management. These factors include neuropathic pain, breakthrough pain, psychological distress, history of addiction, tolerance, predisposition to side-effects, genetic factors etc. Another limitation is the lack of consensus on the cancer pain outcomes, e.g., definition of adequate pain control, tolerance or opioid dose escalation, final opioid doses, use of adjuvant medications, non-pharmacological modalities, and the role of interdisciplinary healthcare professionals.[4] These factors along with the diverse interpretations of the pain experience complicate the development of a standardized pain classification system.

Measurement of pain has remained a burning issue for decades and there is still no gold standard method for evaluating pain. Commonly used methods to measure pain include Visual Analogue Scale (VAS), 101-point Numerical Rating Scale (NRS-101), 11-point Box Scale (BS-11), 6-point Behavioral Rating Scale (BRS-6), 4-Point Verbal Rating Scale (VRS-6) etc. The Edmonton Classification system for Cancer Pain (ECS-CP) recently attempted to classify cancer pain on the basis of seven characteristics having clinical prognostic value. The characteristics included mechanism of pain, incidental pain, daily opioid use, cognitive function, psychological distress, tolerance, and a past history of addiction or alcoholism. Depending on the combinations of these features, patients were defined as having good, intermittent or poor prognosis for pain control.[4]

Management of pain involves efforts from all the healthcare providers engaged in the curative and palliative phase of treatment but maintaining this continuity while the patient shifts from one phase to another is the biggest challenge. The transition from the curative to the palliative phase represents a critical juncture at which continuity of care is often compromised. Therefore, it is necessary that this transition should take place gradually.[5] The challenge of maintaining continuity grows progressively as the number of professionals involved with a patient increases.

Palliative radiotherapy (PRT) is indicated in 30-50% of all cancer patients and patients receiving PRT should be adequately attended for pain and symptom relief. Issues related to pain, difficult physical symptoms and psychosocial aspects can coexist during PRT, which needs to be addressed by a palliative care unit (PCU).

The term palliative care has often been misused; many people still refer to palliative medicine as being entirely focused on terminal illness.[6] Palliative care specialists are faced with extensive barriers to providing effective end-of-life care. It is important to explore these barriers to PCU and hospice referrals because late referrals result in low family satisfaction and adverse clinical outcomes. Many studies have identified barriers like difficulty in predicting prognosis, physician's unwillingness to refer, physician's unfamiliarity with hospice, physician's negative opinion of hospice services, a medical system that does not include hospice as standard care, lack of acceptance of terminal diagnosis by the patient and his family, patient and his family's unwillingness to use hospice and their desire for life-prolonging treatment, insufficient knowledge amongst general population about hospice service, and social attitudes towards health.[7]

In addition, there are other PCU-related issues which include poor access to PCUs (shortage of PCUs, inconvenient locations), environment of PCUs (private room, loneliness, and isolation from general ward), poor communication between staff of PCU and general ward, economic problems (expensive room fees and hospital bills), doctrine beliefs of PCU (emphasis on philosophy, stringent rules for admission) etc.[7]

The aim of the present study was to assess the pain management and referral trend from the radiotherapy department to the pain clinic, PCU or the hospice in a tertiary multidisciplinary academic centre. The study also evaluated the characteristics of pain assessment, management and follow-up in the pain clinic of those patients who actually were seen in the in-house pain clinic and PCU. The objective was to assess the shortcomings and difficulties that prevail in such a scenario so that adequate steps can be taken in the future to improve the overall pattern of referral, assessment, treatment and follow-up of cancer patients who are having pain and need palliative care.

## MATERIALS AND METHODS

The present study is a retrospective analysis done in the Radiation Oncology department of Dr. B R Ambedkar Institute Rotary Cancer Hospital (IRCH), All India Institute of Medical Sciences (AIIMS), New Delhi, India. AIIMS is a multidisciplinary biomedical institution and IRCH is the dedicated cancer facility within this center. This hospital is a regional cancer center under the National Cancer Control Program of the Government of India for patient care, academics and cancer research. The cancer facility has an in-house pain clinic (running Out Patient Department OPfour days a week) and a PCU with six beds. A freestanding hospice, Shanti Avedna Sadan, exists within a distance of 1.5 kilometers from the AIIMS campus, which provides free care to deserving patients.

Overall, 152 consecutive patients, whose files with reasonable treatment details were available, were studied irrespective of the age, sex, primary and consultant in charge of the case. All these patients received PRT in our department between October 2006 and August 2008. The following information was retrieved from their IRCH file in each case: Age at diagnosis, gender, main symptom and its duration, location of primary, disease extent, pain assessment, treatment outcome, referrals and compliance to it, status at last follow- up, and follow-up duration. The referral trend was analyzed in the form of total number of patients who presented with pain and those who were referred to the pain clinic of IRCH, number of patients who reached pain clinic and the number of patients who defaulted.

Patients of all types of advanced malignancies irrespective of their primaries were included in the study except those with bone metastases. Patients with bone metastases were excluded as it formed a major proportion of the patients with advanced cancer and their inclusion could have introduced a sampling bias in the study.

The need for referral was assessed according to the status of the patient on the last day of PRT. The pain clinic details were obtained from the pain chart attached in the file where the pain assessment was done on an 11-point scale (0-10) with 0 representing “no pain at all” and 10 representing the “most severe pain that can be imagined”. Duration of follow-up (DOFU) was defined as the date of last follow-up or death minus date of diagnosis and it was used as a surrogate parameter to estimate overall survival of the patient since data regarding patient's death was not available in most cases.

## RESULTS

### Patient's characteristics

In the group of 152 patients evaluated in our study, there were 119 (78%) males and 33 (22%) females with a median age of 55 years (range 1-86 years). Most of the primaries were of head and neck region (60%) followed by gastrointestinal malignancies in 14% and lung cancer in 11% cases. As expected, most of the cases, i.e. 72% belonged to Stage IV at presentation, followed by 27% cases in Stage III. In two cases, stage was not mentioned in the record. Overall, metastases were present in 24 (16%) cases with lung as the commonest site of metastases (37% cases) followed by brain (29% cases) and liver [Table 1].

**Table 1:** Patient characteristics

Characteristic	Number (percentage)
Total patients	152
Age (in years)	
Median	55
Range	1-86
Sex	
Male	119 (78)
Female	33 (22)
Primary site	
Head and neck	92 (60)
Gastrointestinal tract	21 (14)
Lung	16 (11)
Gynecological	10 (06)
Others	12 (08)
Unknown	01 (≤1)
Stage	
III	41 (27)
IV	109 (72)
Unknown	02 (≤2)
Metastases	
Total cases	24 (16)
Lung	09
Brain	07
Liver	05
Bone	03

### Treatment characteristics

Out of total 152 cases, only 46 (30%) cases had received cancer-directed therapy in the past with chemotherapy being the commonest used modality. All other cases were considered for PRT upfront, either in view of advanced disease at presentation or due to the poor performance status of the patient. The commonest indication for PRT was cytostatic PRT (39% cases) in the presence of progressive visible growth or ulceration. The intention in those cases was either relieving the pressure symptoms due to tumor mass or to control the bleeding, discharge or pain due to ulceration The second commonest symptom for which palliation was considered was dysphagia in 52 (34%) patients. Dyspnea was seen in 19 (12.5%) patients and pain as the primary symptom for palliation was present only in 13 (08%) patients [Table 2]. The PRT dose ranged from 8-30 Gy delivered in one to 10 fractions over the duration of one to 14 days.

**Table 2:** Treatment characteristics

Characteristic	Number (percentage) n = 152
Cancer-directed therapy	
None	106 (70)
RT only	02 (1.3)
CT	23 (15)
Surgery	12 (08)
Surgery + CT	07 (05)
RT + CT	01 (≤1)
Indication for PRT	
Cytostatic	59 (39)
Dysphagia	52 (34)
Respiratory	19 (12)
Pain	13 (08)
Bleeding	07 (05)
Raised intracranial tension	02 (≤2)

CT = chemotherapy; RT = radiotherapy

### Referral pattern and pain characteristics

Out of total 152 cases, 93 cases (61%) were having some degree of pain at the assessment done during the period of PRT or on the day of RT completion. In three patients no details were found regarding pain while in the remaining 56 (37%) cases, no pain was reported by the patient. The severity of pain according to any assessment scale was not mentioned by the radiation oncologist in most of the files.

Out of the 93 patients with pain, 33 patients (35%) were not referred to the pain clinic. Referral details of four patients were not found in the file, while the remaining 56 (61%) patients were referred to the pain clinic at IRCH. All except one patient, who were referred to the pain clinic actually followed the instructions and were later seen in the pain clinic. Only five patients out of the total 152 cases were referred to hospice care [Table 3].

**Table 3:** Referral pattern and pain characteristics

Characteristics	Number (percentage)
Referral pattern (n = 93)	
Not referred to PC	28 (30)
Referred and followed in PC	55 (60)
Referred but defaulted	01 (01)
Unknown	04 (04)
Referred for hospice care	05 (05)
Pain score (n = 55)	
Median	8
3-5	09 (16)
6-8	33 (60)
9-10	13 (24)
Pain treatment (n = 55)	
Step 1	02 (3.5)
Step 2	19 (34.5)
Step 3	34 (62)
Pain relief characteristic (n = 55)	
Adequate pain relief	18 (33)
Partial pain relief	14 (25)
Progressive pain	01 (02)
Unknown status	22 (40)

PC = Pain clinic

In the pain clinic, patient's pain was assessed on the 11-point pain assessment scale. In the 55 patients assessed in the pain clinic, the median pain score was found to be 8, with a range of 3-10. Thirty-one (20%) of patients had a score of 8 or more and were categorized to have severe pain.

When patient's pain treatment was analyzed with respect to the WHO ladder of pain management, it was seen that 62% patients were treated up to Step 3 of the ladder i.e. strong opioids were ultimately used in them. About 35% patients were managed with Step 2 and less than 4% were treated with Step 1 of the WHO ladder [Table 3]. Most of the patients were started on Step 2 upfront in the pain clinic as they were already taking one or the other Step-1 analgesics prescribed in advance of attending the pain clinic.

### Treatment results and follow-up pattern

Out of the 55 patients treated at the pain clinic, 18 patients (33%) achieved adequate pain relief and 14 patients (25%) had partial relief while one patient had progressive pain in spite of using the strong opioids in increasing doses. In a large number of patients i.e. 40% of cases, the result of analgesic treatment could not be retrieved due to incomplete details in the patients' records [Table 4].

**Table 4:** Follow-up pattern

Characteristic	Number (percentage) n = 152
Status at last follow-up	
Complete relief	06 (04)
Partial relief	40 (26)
Stable status	36 (24)
Progression	25 (16)
Dead	01 (<1)
Lost to follow-up/unknown	44 (29)
Duration of follow-up	
Mean	28.8 weeks
Median	20.8 weeks
Minimum	5 days
Maximum	230 weeks
Further RT planned	22 (14)

At the last follow-up in the hospital, six (04%) patients had complete relief of their overall symptoms following PRT and supportive management, while 26 (40%) patients had partial relief. Progression of symptoms in spite of PRT, analgesics and supportive treatment was seen in 25% cases while in others the symptom score remained constant. One patient expired in the hospital during palliative treatment while the largest proportion of the patients i.e. 44% was lost to follow-up after one or more visits to the radiotherapy clinic or pain clinic. Except one patient, no details regarding patients' death were found in their records.

The median duration of follow-up was 20.8 weeks with a range from as short as five days to as long as 230 weeks.

In 22 cases who received PRT, a very good response to PRT was observed during their follow-up in the clinic and they were considered for further RT till the radical doses [Table 4].

## DISCUSSION

This study needs to be seen with caution as it is retrospective in nature and was done in a referral center of an apex institution, where pain clinic and PCU are available in-house. The scenario is not similar in most other centers in India where such facilities are not available. Moreover, the total number of patients needs to be higher to reach to any definite conclusion. However, this study clearly shows the overall pattern of palliative care and referral trend in such a cancer center.

In our study, a disproportionately large number of male patients was included which could be largely due to the maximum cases being of head and neck region, which is the commonest cancer among males in India. The other contributory factors may be the socio-cultural importance of males being the earning member sin the family.

As expected, about three-fourth of our cases belonged to Stage IV disease which is quite common in the patients receiving palliative treatment.

Overall, the incidence of pain was observed in 61% of our cases, which is consistent with the incidence of pain in the advanced cancer patients reported in the literature. In the medical notes recorded by radiation oncology physicians, there was no mention of the severity of pain in any objective form. This makes the overall assessment of pain impossible and audit difficult. So there is a need to sensitize physicians regarding the importance of proper documentation and to make them familiar with the pain assessment scales and charts.

Out of the total patients who had pain, 30% were not referred to the pain clinic. One reason for this may be the prescription of analgesic treatment by the radiation oncologist himself in cases with mild pain. But this cannot be assumed in the absence of any documentary proof.

One unexpected observation was the excellent compliance of the patients in reporting to the pain clinic after they are advised to do so. Out of the total 56 patients referred to the pain clinic, 55 patients were actually seen in the pain clinic. Thus, it is clear from this study, that if the patient is counseled properly and pain and palliative facilities are available in-house, the dropout rate during transition from the definitive to the palliative phase can be reduced significantly.

The referral to the hospice was very low among our patients (< 5%). This may be due to the existence of in-house PCU or due to under-documentation of these referrals because most of these referrals are made from the central casualty (when patient presents with some terminal emergency) where the patient's record file is not available for putting notes when such decisions are made. The overall follow-up and death recording was suboptimal with 29% of the patients being lost to follow-up or having unknown status at the last follow-up. Only one patient had his death documented in the hospital records.

The median pain score was 8. There were very few patients with a pain score of 5 or less. This again may be due to the patients with mild pain being managed by radiation oncologists themselves. The opioids were liberally used in our patients in the pain clinic with 96% of the cases being prescribed some form of the opioid. This was due to the availability of these drugs in the in-house pharmacy, which is a key factor in optimizing the use of opioids. But, in spite of the high use of opioids, only 30% cases experienced adequate pain relief. In as many as 40% cases, the response to analgesic treatment was not documented properly. This observation clearly indicates the importance of proper assessment and follow-up of patients who are started on opioids so that their doses can be titrated properly. In a large study from Netherlands, over 1400 cancer patients were surveyed in five hospitals and the prevalence of cancer pain was 55%, VAS > 4 was 25% and according to the pain management index, it was inefficiently treated in 42% of all patients.[3] Unfortunately, our results in the present study are similar to these reports.

Overall, the findings of our study indicate a clear need for accurate recording of pain and related symptoms as an essential part of the management of patients receiving palliative oncological treatment. These results suggest that although the standard of pain management was satisfactory at our center, we need to improve upon the proper assessment and follow-up of our patients.

## What is the big problem?

Although the idea of PCUs was initially conceived to provide compassionate care, helping patients die peacefully and with dignity, providing care for families, and for alleviating pain, there are several misconceptions still prevailing regarding these institutions. Various studies have suggested that a negative image of the PCU and hospice is recognized as the most important barrier by patients, families, and medical staff.[7] They are described as a place of death and it is thought that once a patient is admitted in the PCU, he or she will never be discharged alive. It is also believed that the PCU shortens the patient's life, isolates him from the community, and does not offer medical treatment.

It is evident from various reports that continuity of care has proven to be a major challenge in comprehensive cancer care, with a number of shortcomings. Oncology physician is often not informed when patients are discharged from the PCUs. This break in the informational continuity has an adverse effect on the quality of follow-up.

In addition, the data appearing in medical files is often primarily of medical nature, with psychosocial issues often being less well documented.[5]

Current training in palliative oncology seems inadequate in terms of providing comprehensive care. Caregivers are often ill-prepared for confronting the experience of death, and providing supportive care and attention to a patient with advanced cancer. Education in India is good, but information alone may not be sufficient to change the physician's attitude and practice.

There is lack on the part of institutions in providing protocols regarding referral guidelines for patients who require pain clinic, PCU or hospice referral. These issues being subjective in nature and often influenced by the clinical judgment and socio-cultural background of an individual physician, need to be considered with utmost care.

There is a paucity of well-designed studies in the literature regarding referral patterns and trends of cancer patients during their advanced disease. Many attempts at conducting performing well-designed studies have failed. The literature is littered with poor or failed studies. Specific challenges include difficulty in recruiting patients, small samples, higher attrition rates, rapidly changing clinical situations, limited duration of survival and gate-keeping by the healthcare professionals.[8] Gate-keeping is the reluctance on the part of primary healthcare professionals to contribute patients for research studies.

Studies to date have suggested that if family caregivers are not adequately prepared for their role in controlling their relative's pain, it may never be possible to achieve the ultimate goal of palliative care. Thus, considerable potential may exist for improving pain management outcomes by supporting and developing caregivers' capacity to be involved in their relative's pain management.[9]

## What do we need now?

It would be relevant to develop more efficient mechanisms for transferring information amongst healthcare professionals so as to facilitate the coordination of care and to ensure its continuity. Beaulieu proposed setting up an effective, formal, shared file system to ensure that all the information regarding a patient can be shared amongst his caregivers.[5] In the years to come, the use of online electronic patient files by caregivers of different departments would make it possible to share the information in a more effective way.

The use of a case manager (or “navigator”) is another alternative that could improve the transfer of information. The case manager would serve as a central resource of information. By taking charge of the patient's file, he (nurse, social worker, doctor, or other) would supervise patient care over the course of the disease and would be able to ensure better continuity of care.[5]

In order to enhance appropriate patient follow-up, care delivery system must be carefully and logically structured. Promoting the in-house pain clinics and PCUs may be of great importance in this regard. Establishing hospices in close proximity to comprehensive cancer care centers with dedicated beds and logistic support (ambulances, minimal paperwork) can improve the compliance of the patients.

A smooth, gradual approach helps to better prepare patients for the shift to the palliative phase of their illness by reducing their fear and anxiety. Nowhere should the referral from one phase to the other be abrupt and sudden. Healthcare professionals can encourage patients to express their feelings and give them the opportunity, wherever possible, to meet the palliative care team before such a transition is made.

When dealing with problems related to the referral process, various authors have suggested formalizing referral protocols between members of the care team. This approach will reduce the ambiguity and confusion encountered while the decision regarding a patient's referral is taken. A well-designed protocol which is accessible to all the residents, nursing staff and other hospital staff should be available so that patients can be guided from time to time with min To ensure the quality of referral trends and palliative care, a localized, reliable, and efficient quality audit tool for clinical practice is greatly needed. These quality audits can serve to monitor, evaluate, and ultimately improve the quality of care.[10]

Several authors have suggested implementing more comprehensive training programs for caregivers by organizing workshops, conferences, seminars etc to improve their skills in delivering care to the advanced cancer patients. Less experienced colleagues should be supported by the superiors or the pain services.

It is important to encourage well-designed studies involving issues in palliative oncology. It is important that the concerns of healthcare professionals are identified and addressed to improve trial recruitment and subsequently patient care.

## CONCLUSION

Various authors have recognized the importance of continuity of care for cancer patients during various phases of their treatment. While it is important to gain insight into these problems, it would also be useful to understand how such problems have arisen over time. A major problem is the attitude of the patients, physicians, and the society as a whole toward cancer pain. Hence, we need to intervene at various levels to solve this problem. At the national level, the solution includes information campaigns to oppose the patient's and physician's “opiophobia”, and to relax our legal intricacies to enhance the availability of morphine and other opioids. On the institutional level, clear policies must be promoted for better pain management. Institutions should promote the use of graphic tools to make pain visible. At an individual level we need to change our attitude towards cancer pain and palliative care.

To correct the misconception prevailing in the society regarding PCUs and hospices, it is important to disseminate accurate information to the general population, patients, and families.
